# The longitudinal volumetric and shape changes of subcortical nuclei in Parkinson’s disease

**DOI:** 10.1038/s41598-024-58187-4

**Published:** 2024-03-29

**Authors:** Wenyi Yang, Xueqin Bai, Xiaojun Guan, Cheng Zhou, Tao Guo, Jingjing Wu, Xiaojun Xu, Minming Zhang, Baorong Zhang, Jiali Pu, Jun Tian

**Affiliations:** 1 Department of Neurology, Second Affiliated Hospital, College of Medicine, Zhejiang University, Hangzhou, 310009 Zhejiang People’s Republic of China; 2 Department of Radiology, Second Affiliated Hospital, College of Medicine, Zhejiang University, Hangzhou, 310009 Zhejiang People’s Republic of China

**Keywords:** Parkinson’s disease, Magnetic resonance imaging, Subcortical nuclei, Putamen, Pallidum, Neurological disorders, Parkinson's disease

## Abstract

Brain structural changes in Parkinson’s disease (PD) are progressive throughout the disease course. Changes in surface morphology with disease progression remain unclear. This study aimed to assess the volumetric and shape changes of the subcortical nuclei during disease progression and explore their association with clinical symptoms. Thirty-four patients and 32 healthy controls were enrolled. The global volume and shape of the subcortical nuclei were compared between patients and controls at baseline. The volume and shape changes of the subcortical nuclei were also explored between baseline and 2 years of follow-up. Association analysis was performed between the volume of subcortical structures and clinical symptoms. In patients with PD, there were significantly atrophied areas in the left pallidum and left putamen, while in healthy controls, the right putamen was dilated compared to baseline. The local morphology of the left pallidum was correlated with Mini Mental State Examination scores. The left putamen shape variation was negatively correlated with changes in Unified Parkinson’s Disease Rating Scale PART III scores. Local morphological atrophy of the putamen and pallidum is an important pathophysiological change in the development of PD, and is associated with motor symptoms and cognitive status in patients with PD.

## Introduction

Parkinson's disease (PD), the second most prevalent neurodegenerative disease, is clinically characterized by bradykinesia, rigidity, and resting tremors. The pathophysiology of PD is the loss of dopaminergic neurons in the substantia nigra (SN), resulting in the dysfunction and structural modification of basal ganglia-thalamocortical circuits modulated by dopamine^1^. Several studies have reported total volume changes in the subcortical nuclei of patients with PD, mainly in the caudate and putamen^2^. However, contrasting results showed no significant differences^3,4^, suggesting that volume changes in subcortical structures were subtle and traditional voxel-based morphometry (VBM) analysis was not sensitive enough to detect them^5^. Techniques aimed at capturing local morphological changes, such as shape analysis, may be more sensitive to such modifications.

Shape analysis, based on vertex analysis, is performed using the FMRIB Software Library (FSL) package and is increasingly used in subcortical studies of neurological disorders. The FMRIB-integrated registration and segmentation tool (FIRST) is an automated tool that has shown high test–retest reliability for the segmentation of subcortical nuclei, especially in the striatum. Moreover, shape analysis can provide more information on subregion deformation, suggesting impairments in different functional networks. In order to correspond to the "global volume" of the nucleus as indicated by the volume measurement, the surface morphology of the local region is hereinafter referred to as "local volume". Studies focusing on local volume differences in subcortical structures have revealed local atrophy in the pallidum^6^, caudate^7^, and putamen^7,8^ compared with healthy controls using shape analysis. However, these studies involved different populations and yielded inconsistent results, suggesting that changes in structural volume vary at different disease stages. Previous findings using VBM have also demonstrated that structural changes are progressive throughout the disease and affect specific subcortical structure^9,10^. Remarkably, Caligiuri et al.^11^ found an extension from the medial to the lateral surface of the putamen in patients with bilateral uptake reduction compared to those with unilateral uptake. The authors hypothesized that the putamen would be damaged in the form of a medial-to-lateral extension as the disease progressed. However, few longitudinal studies have been conducted yet to investigate the shape differences in subcortical structures. Nicholas et al.^12^ reported that local inflation in the thalamus is associated with freezing of gait (FOG) in PD, corresponding to the information processing function of the thalamus in the cortico-basal ganglia-thalamo-cortical circuits. This study investigated the local volume changes in the subcortical nuclei closely correlated with PD (caudate nucleus, pallidum, putamen, and thalamus) during the progression of the disease. In addition, previous studies found that the volume of subcortical structures was correlated with the symptoms of PD^13,14^ but very little was certain^9^. Therefore, the second objective was to clarify the possible association between subcortical structure volumes and clinical symptoms. We aimed to identify regional volume changes in PD during disease progression, and whether these changes are related to the severity of motor or non-motor symptoms.

## Results

### Participants

Thirty-four patients with PD and 32 HCs were recruited for this study. The demographic and clinical characteristics of the participants are shown in Table 1. There were no significant differences in age or sex between the groups. HCs had more years of education than the patients. The HAMD score was higher at baseline in patients with PD than in HCs. The average interval between two scans was 2.08 ± 0.20 years and 2.04 ± 0.24 years for patients with PD and HCs, respectively. The daily levodopa equivalent dose (LEDD) and UPDRS scores (total score, PART II, and PART IV) of PD patients increased over time. At baseline, 4 patients were drug-naïve, 3 patients were treated with levodopa only, and 27 patients received multicomponent medication therapy. At follow-up, 5 patients were treated with levodopa only, and 29 patients received multicomponent medication therapy. In these patients, 2 was taking razagiline, which is reported to has a possible disease-modifying effect in PD^15^, regularly at follow-up.

**Table 1:** Demographic and clinical data of PD patients and HCs.

	PD Patients (n = 34, mean ± SD)		HCs (n = 32, mean ± SD)		p^a,^^b^	p^c^	p^d^
	Baseline	Follow-up	Baseline	Follow-up			
Age	59.43 ± 8.62	–	61.32 ± 7.16	–	0.336	–	–
Gender (F/M)	14/20	–	20/12	–	0.086	–	–
Education	7.68 ± 4.86	–	11.25 ± 3.37	–	0.010*	–	–
Duration	2.39 ± 1.90	4.52 ± 1.86	–	–	–	–	–
TIV	1484.21 ± 151.13	1478.38 ± 152.012	1428.44 ± 110.83	1414.07 ± 109.09	0.094	0.047*	< 0.001**
LEDD	233.82 ± 256.28	457.49 ± 302.25	–	–	–	0.001**	–
H-Y stage	2.09 ± 0.66	2.16 ± 0.40	–	–	–	0.419	–
UPDRS total	27.18 ± 17.44	33.35 ± 18.02	–	–	–	0.049*	–
UPDRS part I	1.21 ± 1.43	1.50 ± 1.29	–	–	–	0.244	–
UPDRS part II	6.56 ± 4.67	8.88 ± 5.56	–	–	–	0.008**	–
UPDRS part III	18.68 ± 12.24	21.76 ± 12.46	–	–	–	0.170	–
UPDRS part IV	0.44 ± 0.75	1.21 ± 1.47	–	–	–	0.007**	–
MMSE	27.44 ± 3.56	26.71 ± 3.51	28.53 ± 1.39	28.13 ± 1.68	0.110	0.088	0.141
HAMD	4.85 ± 3.87	5.24 ± 5.10	2.72 ± 2.89	3.72 ± 5.08	0.014*	0.658	0.114
HAMA	4.79 ± 4.36	5.44 ± 4.85	4.28 ± 4.27	4.72 ± 5.54	0.631	0.516	0.569
PDQ-39	20.79 ± 19.56	24.29 ± 24.96	–	–	–	0.210	–

*LEDD* daily levodopa equivalent dose, *SD* standard deviation.

^a^Unpaired t-test between PD patients and HCs at baseline.

^b^χ2 Likelihood Ratio in gender between PD patients and HCs at baseline.

^c^Paired t-test between PD patients (baseline) and PD patients (follow-up).

^d^Paired t-test between HCs (baseline) and HCs (follow-up).

*p < 0.05. **p < 0.01. The P-values were FDR-adjusted.

The moderate/severe patients had a longer course of disease and higher UPDRS (total score, PART II, PART III) and PDQ-39 scores than the early-stage patients (Table 2).

**Table 2:** Demographic and clinical data of PD patients at different stage.

	Mild (n = 17)	Moderate/severe (n = 17)	p value
Age	57.72 ± 9.51	61.13 ± 7.53	0.256
Gender (F/M)	7/10	7/10	1.000
Education	8.35 ± 5.35	7.00 ± 4.37	0.425
Duration	1.72 ± 1.40	3.06 ± 2.14	0.039*
TIV	1508.90 ± 150.45	1459.51 ± 152.23	0.349
LEDD	147.06 ± 145.47	320.59 ± 313.53	0.050*
UPDRS total	18.29 ± 16.71	35.47 ± 12.89	0.002**
UPDRS part I	0.82 ± 1.13	1.59 ± 1.62	0.121
UPDRS part II	4.41 ± 4.11	8.71 ± 4.28	0.005**
UPDRS part III	12.82 ± 12.67	24.53 ± 8.71	0.004**
UPDRS part IV	0.24 ± 0.44	0.65 ± 0.93	0.113
MMSE	28.00 ± 2.18	26.88 ± 4.56	0.368
MoCA	22.41 ± 5.20	22.06 ± 5.27	0.845
HAMD	5.00 ± 3.89	4.71 ± 3.97	0.829
HAMA	4.35 ± 2.78	5.24 ± 5.57	0.563
PDQ-39	13.00 ± 7.37	28.59 ± 24.61	0.022*

*p < 0.05. **p < 0.01.

### Global and local volume at baseline

There were no differences between patients with PD and HCs in terms of global or local volumes (Table 3 and Supplementary Table S1).

**Table 3:** Global volumes in PD patients and HCs.

Structure	PD patients (n = 34, mean ± SD)			HCs (n = 32, mean ± SD)			p^c^
	Baseline	Follow-up	p^a^	Baseline	Follow-up	p^b^	
Left caudate	3447.92 ± 464.09	3399.10 ± 504.78	0.128	3391.04 ± 355.91	3343.50 ± 422.60	0.469	0.850
Right caudate	3508.68 ± 485.36	3471.22 ± 504.28	0.125	3444.38 ± 440.18	3421.56 ± 411.87	0.469	0.850
Left pallidum	1900.46 ± 225.15	1873.34 ± 224.52	0.125	1887.19 ± 275.30	1888.59 ± 285.47	0.939	0.826
Right pallidum	1863.70 ± 198.70	1859.07 ± 200.71	0.827	1806.62 ± 195.25	1807.98 ± 213.13	0.939	0.850
Left putamen	4862.25 ± 610.10	4795.41 ± 667.05	0.125	4825.53 ± 479.80	4842.78 ± 550.47	0.939	0.826
Right putamen	4774.19 ± 584.03	4728.68 ± 641.03	0.382	4689.69 ± 505.38	4728.79 ± 512.30	0.469	0.826
Left thalamus	7978.53 ± 733.48	7974.82 ± 702.55	0.951	7806.80 ± 632.79	7795.97 ± 580.59	0.939	0.850
Right thalamus	7817.62 ± 684.83	7788.43 ± 683.82	0.827	7684.96 ± 601.62	7643.58 ± 619.47	0.800	0.826

^a^^,b^Paired t-test between baseline and follow-up.

^c^Group comparison between PD patients ang HCs at baseline using general linear model.

*p < 0.05. **p < 0.01. All P-values were FDR-adjusted.

No significant differences were found in global volume of subcortical nuclei between the moderate/severe PD patients and mild patients (Table 4). The moderate/severe PD patients showed local shape atrophy in left putamen compared to mild patients, but the p-value did not indicate significance when multiple comparison correction was performed (Supplementary Table S2).

**Table 4:** Global volumes of the subcortical structures in different PD stages.

Structure	Mild	Moderate/severe	p value
Left caudate	3529.94 ± 435.57	3364.37 ± 491.75	0.937
Right caudate	3580.18 ± 447.80	3450.21 ± 511.28	0.937
Left pallidum	1921.40 ± 201.82	1921.32 ± 293.51	0.937
Right pallidum	1874.87 ± 211.36	1876.55 ± 209.11	0.937
Left putamen	5054.12 ± 654.77	4700.83 ± 524.27	0.937
Right putamen	4855.77 ± 646.74	4699.52 ± 519.30	0.937
Left thalamus	8192.551 ± 692.73	7814.38 ± 672.02	0.937
Right thalamus	7971.29 ± 711.85	7683.55 ± 620.45	0.937

P value was FDR-adjusted. No significant differences were found in global volume between patients in different stages.

### Global and local volume change in PD patients and HCs

**Figure 1:**
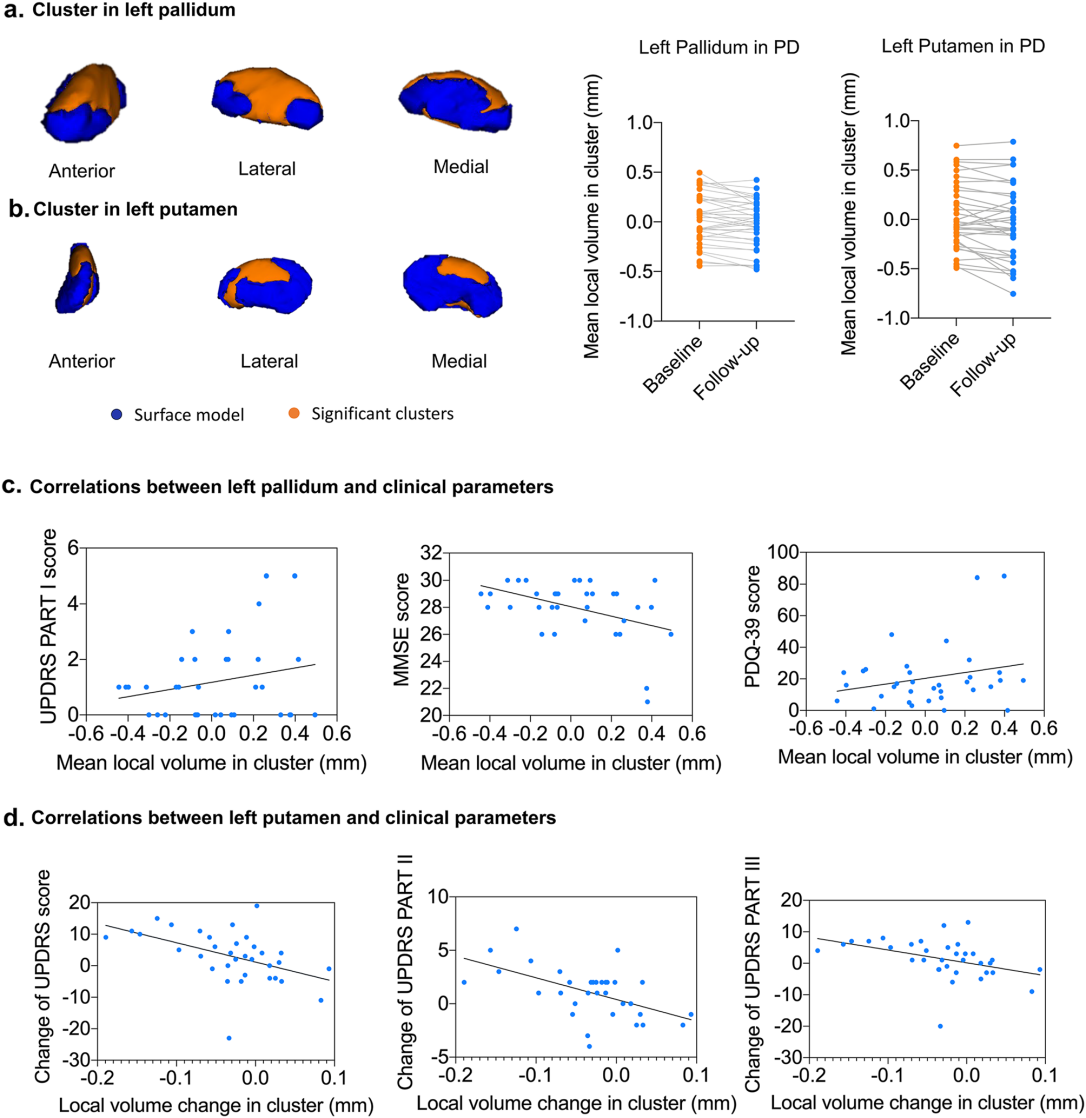
Longitudinal local volume alterations in PD patients over two years. Surface models of subcortical structures are depicted in blue and significant clusters are in orange. Clusters and p values effect from the paired t-test between baseline and 2 years later. (**a**) Significant atrophy in local volume was found in the left pallidum. Post-hoc tests revealed that PD patients showed local inflation over 2 years. (**b**) Significant atrophy in the left putamen over 2 years. (**c**) The mean local volume value of the left pallidum correlated with UPDRS PART I, PDQ-39 and MMSE scores. (**d**) The change rate of the left putamen local volume value correlated with the change in UPDRS total, PART II, and PART III scores.

**Figure 2:**
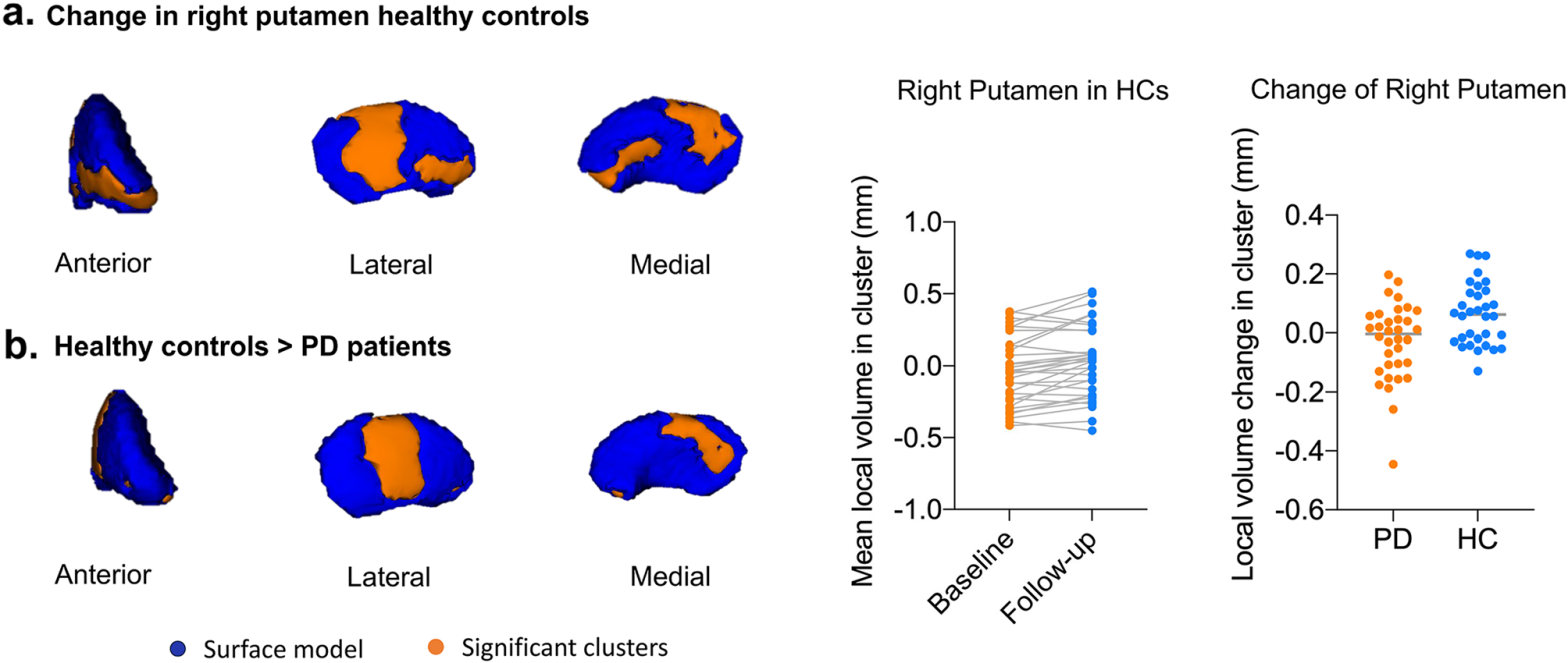
Longitudinal local volume alterations in HCs and different local volume change between PD patients and HCs. (**a**) Significant inflation in local volume was found in the left putamen. (**b**) Significant inflation in the left putamen was greater in HCs than in PD patients.

Longitudinal shape analysis revealed significant local atrophy in the left pallidum (p = 0.008) and left putamen (p = 0.032) in PD patients over two years. Clusters over five voxels are shown in Fig. 1a,b. The mean local volume of each cluster was extracted to perform a paired t-test for post hoc testing (Fig. 1a,b). There was also a cluster that approached significance in the left thalamus (p = 0.051), indicating local deflation (Supplementary Table S3). Meanwhile, significant local inflation was detected in the right putamen (p = 0.008) during the follow-up of HCs (Fig. 2a). In the group comparison, the longitudinal inflation of the right putamen in the HC group was greater than that in the PD group, while patients with PD did not demonstrate significant changes (Fig. 2b). No significant results were found for the global volume (Tables 3, 5).

**Table 5:** Differences in global volumes change of PD Patients and HCs.

Structure	PD patients (n = 34, mean ± SD)	HCs (n = 32, mean ± SD)	p value
Left caudate	− 48.82 ± 148.72	− 47.54 ± 189.85	0.910
Right caudate	− 37.46 ± 102.59	− 22.82 ± 103.02	0.910
Left pallidum	− 27.12 ± 68.24	1.39 ± 101.70	0.539
Right pallidum	− 4.62 ± 75.69	1.35 ± 82.88	0.910
Left putamen	− 66.84 ± 189.10	17.25 ± 273.58	0.236
Right putamen	− 45.50 ± 221.28	39.10 ± 113.87	0.236
Left thalamus	− 3.71 ± 352.82	− 10.83 ± 272.95	0.910
Right thalamus	− 29.20 ± 383.93	− 41.39 ± 274.26	0.910

All P-values were FDR-adjusted.

The mean local volume of the identified clusters was correlated with clinical parameters. The mean local volume value of the left pallidum positively correlated with scores on the UPDRS PART I (p = 0.041, rho = 0.376) and PDQ-39 (p = 0.027, rho = 0.402) but negatively correlated with the MMSE score (p = 0.032, rho = − 0.393) at baseline (Fig. 1c). The change in the local volume of the left putamen was negatively correlated with changes in the UPDRS total (p = 0.012, rho = − 0.454), UPDRS PART II (p = 0.010, rho = − 0.464), and UPDRS III (p = 0.036, rho = − 0.385) scores (Fig. 1d).

## Discussion

This study investigated subcortical structural volume changes in patients with PD over time. At the time of entry into the study, no significant global or local volume alterations were observed between patients with PD and HCs. Local shape atrophy trend in left putamen was detected in moderate/severe PD patients compared to mild patients. The moderate/severe patients had a longer mean duration of disease than mild patients, suggesting that this alteration occurred as the progression of the disease. The longitudinal study also showed local volume decrease in the left putamen in patients with PD, corresponded with the cross-sectional analysis above. Several cross-sectional studies have been conducted on patients and controls regarding shape differences. Nemmi et al.^7^ found local volume atrophy in the medial and anterolateral aspects of the bilateral putamen in treated patients, whereas Lee et al.^8^ found shape alterations in the posterolateral and ventromedial putamen in drug-naïve patients. Another study in drug-naïve patients showed shape atrophy of the medial aspect of the putamen in patients with unilateral abnormal DAT-SPECT and to a greater extent over the anterolateral putamen in patients with bilateral damage, suggesting that the lateral surface is involved later in the course of the disease^11^. The current study revealed the involvement of both the medial and lateral aspects of the putamen, but mostly the lateral aspect, in a treated patient cohort. Therefore, atrophy of the lateral aspect may be altered during the progression of PD. Consistent with the findings of Nemmi et al., local atrophy was observed in the anterolateral region of the putamen. The medial part of the putamen is connected to the premotor area and the lateral surface to the primary motor area^16^. Our study suggests that the primary motor areas involved in the pathogenesis of PD motor symptoms are mainly involved in the progression of the disease. Moreover, clinical parametric analysis revealed a correlation between the decrease in the local volume of the left putamen and the increase in the UPDRS and UPDRS PART II and III scores, suggesting a more rapid progression of motor symptoms. This is consistent with the fact that the putamen is the main motor structure in the striatum^17^. A previous study also demonstrated that local atrophy in the left putamen correlated with the UPDRS right motor score^7^. The larger local volume of anterior was reported to attenuate the increase of LED over time, associated with higher motor compensatory ability in patients with PD^18^.

Previous studies have demonstrated shape atrophy of the right pallidum in patients with PD compared with HCs^6,19^. Vertex-wise analysis also demonstrated extensive local volume atrophy of the left pallidum in patients with PD, mainly on the anterodorsal surface as part of the globus pallidus externus (GPe). The GPe is classically considered a component of the indirect pathway that plays a role in motor inhibition and is overactive in PD^20^. The GPe is composed of rich neural circuitry of diverse cell types that influence motor and nonmotor behaviors^21^. The anterodorsal GPe is considered the associative territory, where microinjections of bicuculline, a GABAergic antagonist, produce hyperactivity and/or attention deficits in monkeys^22,23^. A mouse study demonstrated that divergent GPe neurons are associated with locomotion and reversal learning^24^. Our study found that the mean local volume of the left pallidum was positively correlated with cognitive dysfunction at baseline. A higher UPDRS PART I score also indicated worse cognition or mental state. We hypothesized that this cluster underwent subtle structural compensation of the associative territory at baseline and that the perturbation in the GPe contributed to cognitive dysfunction. During disease progression, the cluster reversed to maladaptation.

A cluster that approached a significance threshold showed local deflation in the left thalamus. The thalamus plays a role in information modulation and integration through its connections with the basal ganglia, cerebellum, and cortex^25,26^. Abnormal structural alterations in the thalamus have been detected in previous studies comparing patients with PD and HCs^27^ and might be associated with FOG in PD^12,28^.

The finding that only the left but not the right subcortical structures showed significant shape differences during the two years may reflect a trend towards higher right UPDRS PART III scores (7.24 ± 6.18 vs. 5.59 ± 5.30, p = 0.196, paired t t-test) for the PD patients at baseline. Furthermore, all the subjects included in our study were right-handed. The dominant hand side is affected more often first in PD and is usually the dominant side of symptoms^29^. The asymmetry of symptoms may correlate with the disruption of the lateralized brain activity pattern^30^.

Significant local inflation was detected in the right putamen of HCs and was found to be greater than in patients with PD. The physiological mechanisms of local increases in gray matter include neuronal and non-neuronal activity-dependent changes, such as those in the vasculature and glial cells. These activities are modulated by neurotransmitters and neurotrophic factors that contribute to structural changes resulting from learning or other experiences^31^. Shape alterations in HCs were seen mostly in the lateral, posteromedial, and anterior putamen, whereas these changes were not observed in patients with PD. Connected to the executive prefrontal regions, the rostral putamen is considered part of the associative network, which plays a role in the integration of sensory and motor activity^17,32^. In healthy individuals, the local volume of the anterior putamen decreases as a function of age, which may be related to an age-related decline in cognitive capabilities^33^. A study focusing on patients with multiple sclerosis revealed that cognitively preserved patients have significantly increased putamen volume compared to cognitively impaired patients^34^. Patients with PD and HCs showed significantly different local shape alterations over two years in the lateral and posteromedial regions of the right putamen, and these areas mainly participated in motor manifestations^16,35^. The increasing volume of clusters may reflect the age-related adaptive nature of healthy individuals, which does not occur in patients with PD.

This is the first longitudinal study to compare morphological changes in patients with PD and HCs using shape analysis. These morphological changes suggest that functional circuits may become dysfunctional during PD development. This study had some limitations. First, the time of follow-up chosen in this study might not represent the entire process of change in the subcortical nuclei. Further research with more follow-up time points is required. Second, the relatively small sample size may have reduced the sensitivity to subtle shape variations. The post-hoc analysis indicated the power in the local volume differences was19.0% in the left pallidum and 17.5% in the left putamen respectively. The relative small sample size and large standard errors may account for the relatively low power. Therefore, studies with large cohorts of patients with PD are meaningful. The heterogeneity of the PD cohort, such as medication status and disease severity. Further researches are needed to study the influence of heterogeneity on local volume in subcortical nuclei.

## Conclusion

In conclusion, the local atrophy of the pallidum and putamen is involved in the progression of PD. Regional atrophy of the left pallidum is associated with cognitive impairment, and changes in the left putamen are associated with the progression of motor symptoms. Because the sample size was relatively small, the study results must be interpreted with caution.

## Methods

### Participants

This study included 34 patients with PD and 32 healthy controls (HCs). All patients were admitted to the Neurology Department of the Second Affiliated Hospital of Zhejiang University and diagnosed by a senior movement disorder specialist^36^. All participants were judged to be right-handed according to the Edinburgh Handedness Inventory. Participants with a history of major medical illness, cerebral trauma, psychiatric or other neurological disorders, or alcohol abuse were excluded. All images were visually checked by an experienced radiologist and rechecked by another radiologist before preprocessing for image quality control. Subjects with severe head movement or other artifacts were excluded from the study. Ethical approval was obtained from the Medical Ethics Committee of the Second Affiliated Hospital of Zhejiang University School of Medicine, and all participants provided written informed consent. The study conforms with World Medical Association Declaration of Helsinki published on the website of the Journal of American Medical Association.

All participants underwent magnetic resonance imaging (MRI) scanning and clinical evaluations at baseline and 18–30 months later; both were carried out while the participants were on an “OFF” period (experiencing at least 12 h of withdrawal of their anti-parkinsonian medication).

### Clinical evaluations

The H-Y Stage was accessed and patients in I–II stage were classified as mild and patients in III–IV stage were classified as moderate/severe^37^. The severity of the movement symptoms was assessed using the Unified Parkinson’s Disease Rating Scale (UPDRS-III). Cognition was assessed using the Mini Mental State Examination (MMSE). The Hamilton Depression Scale (HAMD) and Hamilton Anxiety Scale (HAMA) scores were used to assess depression and anxiety. The Parkinson’s Disease Questionnaire-39 (PDQ-39) was used to evaluate quality of life. Longitudinal clinical score changes were calculated as follows: (score_follow-up_ − score_baseline_)/(time_follow-up_ − time_baseline_).

### MRI data acquisition

MRI data were obtained using the same 3.0 Tesla MRI machine (GE Discovery 750) equipped with an 8-channel head coil. 3D T1-weighted images were acquired using a fast-spoiled gradient-recalled sequence (repetition time, 7.336 ms; echo time, 3.036 ms; inversion time, 450 ms; flip angle, 11°; field of view, 260 × 260 mm^2^ matrix, 256 × 256; slice thickness, 1.2 mm; in-plane resolution, 1 × 1 mm; and 196 continuous sagittal slices). During scanning, the participants were told to close their eyes and remain still, with restraining foam pads applied to stabilize their heads.

### MRI processing

Subcortical segmentation of T1 MRI data was performed using the FIRST, a part of the FMRIB's Software Library (FSL, version 5.0.9, https://fsl.fmrib.ox.ac.uk/fsl/fslwiki/FIRST). Volumetric and mesh images of the subcortical nuclei (both at baseline and at follow-up) were acquired for each participant. For mesh images, a surface mesh of subcortical structures composed of vertices was created using a deformable mesh model^38^. Shape analysis was performed using surface-based per-vertex analysis. The local volume was calculated as the distance of the vertices in each individual from the mean sample vertices to quantify the shape differences on the surface of the nuclei. The global volumes were extracted from the segmented meshes and calculated as the volume within the surface mesh. Longitudinal volumetric changes were calculated as: volume_folllow-up_ − volume_baseline_. Longitudinal local volume changes were calculated as mesh_folllow-up_ − mesh_baseline_^12^.

### Statistical analyses

To analyze global and local volume alterations, we performed both cross-sectional and longitudinal analyses as follows. (1) At study entry, we compared the global and local volumes of subcortical structures between patients with PD and HCs, then between mild and moderate/severe PD patients.. (2) Using change data over two years, we investigated significant global and local volume changes in patients with PD and HCs. (3) We also searched for structural differences showing differential progression by comparing volume variations between patients with PD and HCs.

Total intracranial volume (TIV) was estimated using FreeSurfer (version 6.0). Global volume analysis was performed with the general linear model in analyses (1) and (3), using age, sex, and TIV as covariates. The paired t-test was used for global volume analysis (2). Local volume was analyzed using the FSL’s randomized tool to perform a non-parametric permutation test^39,40^ in (1)–(3). The matrix and t-contrast were created using a general linear model GUI (Glm GUI). In analyses (1) and (3), an analysis of covariance was used with age and sex as nuisance regressors, and paired t-tests were used for analysis (2). Five thousand permutations were performed, and the family wise error (FWE) rate was corrected. The vertices of the clusters (> 5 voxels) showing a significant effect were extracted to calculate the mean local volume, and post-hoc analyses were performed using SPSS v.26 (IBM Corp, NY). False discovery rate (FDR) testing was performed for multiple comparisons, and statistical significance was set at p < 0.05. significant. Local cluster volumes in each participant were calculated, and correlations with clinical parameters were analyzed using Spearman’s partial correlation rho, with age, sex, and education as covariates.

### Ethics approval

Ethical approval was obtained from the Medical Ethics Committee of the Second Affiliated Hospital of Zhejiang University School of Medicine.

### Consent to participate

All participants provided written informed consent. The study conforms with World Medical Association Declaration of Helsinki published on the website of the Journal of American Medical Association.

### Supplementary Information

Supplementary Tables.

## Data Availability

The data that support the findings of this study are available from the corresponding author upon request.
