# Supplementary material for: The longitudinal volumetric and shape changes of subcortical nuclei in Parkinson’s disease
Source: Sci Rep. 2024 Mar 29;14:7494. doi: 10.1038/s41598-024-58187-4 (PMC10980751; doi:10.1038/s41598-024-58187-4)
Supplement: Supplementary file 1 — Supplementary Tables. [file 41598_2024_58187_MOESM1_ESM.docx]

**The** **longitudinal volumetric and shape changes of subcortical nuclei in Parkinson’s disease**

**Wenyi Yang^#1^, Xueqin Bai^#2^, Xiaojun Guan^2^, Cheng Zhou^2^ Tao Guo^2^, Jingjing Wu^2^, Xiaojun Xu^2^, Minming Zhang^2^, Baorong Zhang^1^** **Jiali Pu^1^, Jun Tian*^1^**

**^1^**Department of Neurology, Second Affiliated Hospital, College of Medicine, Zhejiang University, Hangzhou, Zhejiang 310009, China

**^2^**Department of Radiology, Second Affiliated Hospital, College of Medicine, Zhejiang University, Hangzhou, Zhejiang 310009, China

#These authors contributed equally to this work

*Correspondence to:

Jun Tian, M.D., Department of Neurology, Second Affiliated Hospital, College of Medicine, Zhejiang University, Hangzhou, Zhejiang 310009, People’s Republic of China; Phone: +86-571-87784752; FAX: +86-571-87784752; email: juntian@zju.edu.cn

| **Supplementary**  **Table 1 Differences in local volumes of the subcortical structures between PD patients and HCs** | | | | |  |
| --- | --- | --- | --- | --- | --- |
| Structure | p^1^ (PD < HC) | FDR-adjusted p^1^ | p^2^ (PD > HC) | FDR-adjusted p^2^ |  |
| Left Caudate | | 0.281 | 0.562 | 0.437 | 0.819 |
| Right Caudate | | 0.560 | 0.640 | 0.429 | 0.819 |
| Left Pallidum | | 0.424 | 0.640 | 0.433 | 0.819 |
| Right Pallidum | | 0.872 | 0.872 | 0.192 | 0.819 |
| Left Putamen | | 0.140 | 0.562 | 0.887 | 0.887 |
| Right Putamen | | 0.242 | 0.562 | 0.751 | 0.858 |
| Left Thalamus | | 0.481 | 0.640 | 0.512 | 0.819 |
| Right Thalamus | | 0.239 | 0.562 | 0.745 | 0.858 |

P value was FDR-adjusted. p^1^: local volume decreases in PD compared to HCs. p^2^: local volume increases in PD compared to HCs. No significant differences were found in local volume changes between patients and HCs.

| **Supplementary Table 2 Local Volumes of The Subcortical Structures in Different PD Stages** | | | | |
| --- | --- | --- | --- | --- |
| Structure | p^1^(mild > moderate/severe) | FDR-adjusted p^1^ | p^2^ (mild < moderate/severe) | FDR-adjusted p^2^ |
| Left Caudate | 0.054 | 0.120 | 0.514 | 0.999 |
| Right Caudate | 0.132 | 0.132 | 0.942 | 0.999 |
| Left Pallidum | 0.030 | 0.120 | 0.999 | 0.999 |
| Right Pallidum | 0.090 | 0.120 | 0.769 | 0.999 |
| Left Putamen | 0.087 | 0.120 | 0.892 | 0.999 |
| Right Putamen | 0.128 | 0.132 | 0.867 | 0.999 |
| Left Thalamus | 0.037 | 0.120 | 0.918 | 0.999 |
| Right Thalamus | 0.073 | 0.120 | 0.877 | 0.999 |

P value was FDR-adjusted. No significant differences were found in local volume between patients in different stages.

| **Supplementary Table 3 Local volumes of the subcortical structures in PD patients** | | | | |
| --- | --- | --- | --- | --- |
| Structure | p^1^(baseline > follow up) | FDR-adjusted p^1^ | p^2^(baseline < follow up) | FDR-adjusted p^2^ |
| Left Caudate | 0.301 | 0.344 | 0.175 | 0.872 |
| Right Caudate | 0.131 | 0.210 | 0.218 | 0.872 |
| Left Pallidum | 0.001 | 0.008** | 0.988 | 0.988 |
| Right Pallidum | 0.361 | 0.361 | 0.504 | 0.971 |
| Left Putamen | 0.008 | 0.032* | 0.882 | 0.988 |
| Right Putamen | 0.185 | 0.247 | 0.614 | 0.971 |
| Left Thalamus | 0.019 | 0.051 | 0.663 | 0.971 |
| Right Thalamus | 0.126 | 0.210 | 0.728 | 0.971 |

P value was FDR-adjusted. Significant local atrophy was found in left pallidum and left putamen in PD patients over 2 years. There was cluster approaching significance in the left thalamus showed local deflation. *: p<0.05. **: p<0.01.

| **Supplementary Table 4 Clusters atrophy in PD patients over 2 years** | | | | | | | | |
| --- | --- | --- | --- | --- | --- | --- | --- | --- |
| Structure | Voxels | MAX | MAX X (vox) | MAX Y (vox) | MAX Z (vox) | COG X (vox) | COG Y (vox) | COG Z (vox) |
| Left Pallidum | 542 | 0.989 | 109 | 127 | 75 | 111 | 122 | 71.6 |
| Left Putamen | 811 | 0.982 | 112 | 131 | 78 | 115 | 130 | 74 |

The clusters detected significant atrophy and over 5 voxels were shown.

| **Supplementary Table 5 Local volumes of the subcortical structures in HCs** | | | | |
| --- | --- | --- | --- | --- |
| Structure | p^1^(baseline > follow up) | FDR-adjusted p^1^ | p^2^(baseline < follow up) | FDR-adjusted p^2^ |
| Left Caudate | 0.496 | 0.496 | 0.511 | 0.511 |
| Right Caudate | 0.197 | 0.263 | 0.04 | 0.107 |
| Left Pallidum | 0.192 | 0.263 | 0.302 | 0.345 |
| Right Pallidum | 0.423 | 0.483 | 0.106 | 0.212 |
| Left Putamen | 0.181 | 0.263 | 0.223 | 0.297 |
| Right Putamen | 0.08 | 0.263 | <0.001 | 0.008** |
| Left Thalamus | 0.131 | 0.263 | 0.161 | 0.258 |
| Right Thalamus | 0.038 | 0.263 | 0.037 | 0.107 |

P value was FDR-adjusted. Significant local inflation was found in right putamen in HCs over 2 years. **: p<0.01.

| **Supplementary Table 6 Differences in local volumes of the subcortical structures** | | | | |
| --- | --- | --- | --- | --- |
| Structure | p^1^(∆PD < ∆NC) | FDR-adjusted p^1^ | p^2^(∆PD > ∆NC) | FDR-adjusted p^2^ |
| Left Caudate | 0.722 | 0.722 | 0.417 | 0.456 |
| Right Caudate | 0.528 | 0.603 | 0.255 | 0.408 |
| Left Pallidum | 0.094 | 0.221 | 0.444 | 0.456 |
| Right Pallidum | 0.166 | 0.221 | 0.215 | 0.408 |
| Left Putamen | 0.030 | 0.118 | 0.207 | 0.408 |
| Right Putamen | <0.001 | 0.008** | 0.139 | 0.408 |
| Left Thalamus | 0.115 | 0.221 | 0.126 | 0.408 |
| Right Thalamus | 0.165 | 0.221 | 0.456 | 0.456 |

P value was FDR-adjusted. Greater local inflation of right putamen was found in HCs compared to PD patients. **: p<0.01.

| **Supplementary Table 7 Clusters showing different change in right putamen between PD patients and HCs over 2 years** | | | | | | | | |
| --- | --- | --- | --- | --- | --- | --- | --- | --- |
| Cluster Index | Voxels | MAX | MAX X (vox) | MAX Y (vox) | MAX Z (vox) | COG X (vox) | COG Y (vox) | COG Z (vox) |
| 1 | 583 | 0.994 | 59 | 123 | 79 | 61.6 | 122 | 76.3 |
| 2 | 29 | 0.956 | 75 | 135 | 62 | 74.6 | 137 | 62 |
| 3 | 19 | 0.951 | 67 | 140 | 64 | 66.4 | 140 | 64 |

The clusters detected significant atrophy and over 5 voxels were shown.
